# Galactorrhea after Breast Augmentation with Mastopexy: Case Reports and Literature Review

**DOI:** 10.1055/a-2676-4360

**Published:** 2025-11-20

**Authors:** Juraj Payer, Matej Patzelt, Roman Kralik, Michal Makel

**Affiliations:** 1 Renomé Clinic, Prague, Czech Republic; 2 ABClinic, Prague, Czech Republic; 3 Department of Plastic Surgery, University Hospital Královské Vinohrady, Prague, Czech Republic; 4 Fifth Department of Internal Medicine, University Hospital Bratislava, Bratislava, Slovakia; 5 Department of Plastic Surgery, St. Anne's University Hospital, Brno, Czech Republic

**Keywords:** galactorrhea, breast augmentation with mastopexy, case report

## Abstract

Galactorrhea is a rare but well-described complication following aesthetic breast surgery. Augmentation with mastopexy is a procedure that combines breast tissue reduction with volume augmentation; however, only few cases of galactorrhea were described following this procedure. This report concerns three cases of galactorrhea after augmentation with mastopexy provided by two surgeons (J.P. and M.P.). Patients did not report any significant gynecological history, including a history of oral hormonal intake or galactorrhea in the past. All three patients underwent the dual plane breast augmentation together with the Wise-pattern breast lift. In two cases, the first symptoms occurred in the second week, and in one case in the third week after surgery, manifesting as breast pain and swelling. These clinical issues, accompanied by a small amount of white fluid secretion from the vertical scar, scar around nipple-areolar complex, and from the T-junction, were presented to the operating surgeons. Galactorrhea came to consideration after a negative microbiological examination. In two cases, galactorrhea was treated with oral dopaminergic medication cabergoline, with a good response to the therapy. All symptoms, including redness and pain, fully resolved at the 21st postoperative day without the necessity of surgical revision.

One patient with bilateral secretion was treated by early operative revision. In the following days, secretion ceased, and after drain removal, further complications did not occur. Aesthetic results after 12 months were satisfactory in all patients.

In conclusion, galactorrhea is a rare but significant complication of any mammaplasty surgery. Untreated secretion may lead to severe complications; however, early recognition and treatment may lead to good results and patient satisfaction.

## Introduction

Galactorrhea is defined as spontaneous milk secretion from the breast unrelated to pregnancy or lactation. If it occurs within 12 months after cessation of breastfeeding, it is considered physiological. 1 Galactorrhea as a complication after breast surgery occurs rarely; however, it should not be overlooked as it can lead to further complications such as wound dehiscence, necrosis, and infection. 2

**Fig. 1:**
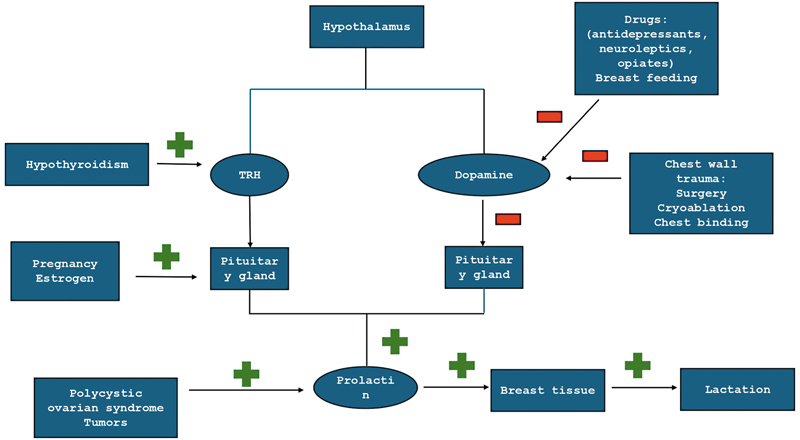
Pathophysiology of galactorrhea.

To recognize and treat postoperative galactorrhea correctly, the physiology of milk production must be understood. Mechanical irritation of the chest wall and surgical manipulation of the breast tissue have been hypothesized to cause galactorrhea. 3 In some cases, galactorrhea may be a symptom of pituitary neoplasm, an endocrine disease, or a side effect of a medication influencing the dopamine system 4 5 ( Fig. 1 ).

Proper treatment should be initiated to prevent further complications. Modern approaches to galactorrhea treatment algorithms involve dopamine agonists being the drugs of choice. According to a recent meta-analysis, cabergoline seems to be the safest option among them. 6

Most of the described cases of galactorrhea in the literature are associated with breast augmentation with prosthetic implants, 3 7 8 9 10 11 12 followed by breast reduction or mastopexy. 2 13 14 15 One staged augmentation with mastopexy has gained popularity in recent years and nowadays belongs to the standard portfolio of plastic surgeons. This surgical technique is a combination of breast tissue reduction and juxtaposed nipple–areolar complex (NAC) reposition with breast volume augmentation. 16 Although mastopexy augmentation is considered to be a safe procedure in the hands of experienced surgeons with an estimated complication rate of 1.86%, 17 it still is a combination of two opposing surgical techniques and thus shows overall higher complication rates than mastopexy or augmentation alone. 18 Despite this, there are only very few cases describing galactorrhea following this procedure. 7 19

So far, published studies about galactorrhea as a complication of cosmetic breast surgery have mostly been case reports 2 7 10 11 12 13 14 15 20 and retrospective studies, 8 and the long-term aesthetic outcomes have only been described sporadically.

The aim of our study was to present three clinical cases of galactorrhea following augmentation mastopexy with long-term follow-up and compare our results with the existing literature.

### Pathophysiology

The cause of galactorrhea can vary as it may result from a pathological process in any of the different steps of the physiological axis of prolactin secretion. Prolactin is the main hormone responsible for lactation, and its release is caused by a decrease in prolactin-inhibitory hormone (dopamine) levels in the pituitary gland. This dopamine blood level drop can be caused by central or peripheral stimulation, which occurs naturally during the childbirth period.

Central stimulation can be influenced by hypothalamic–pituitary disorders. Hypothyroidism caused by decreased thyroid hormone in pituitary gland increases thyroid-releasing hormone production, which in turn leads to increased prolactin levels and causes galactorrhea. 21 Correction of hypothyroidism with levothyroxine administration leads to normalization of serum prolactin levels. 22 Dopaminergic antidepressant drugs may also lead to galactorrhea as they work as dopamine antagonists. 1 Peripheral stimulation, such as chest wall irritation, nipple and mammary gland manipulation, and estrogen and progesterone hormone intake, also increases prolactin blood level.

The most probable cause of galactorrhea in our cases was direct chest wall irritation as well as nipple manipulation during breast tissue reduction and incision performed through the glandular tissue. Chest wall irritation may cause decrease of dopamine levels via spinal cord as it mimics nipple stimulation. This theory is supported by reports of patients after severe burns and chest wall injuries presenting with galactorrhea. 23

Risk factors for the development of postoperative galactorrhea after breast surgery include oral contraceptive use, previous pregnancies, hypothyroidism, periareolar incisions, and implant placement below the pectoralis major muscle. 9 11 12 14 24

## Case

### Patients and Methods

As part of the preoperative examination, information was collected on parity, oral contraceptives intake, endocrine diseases, history of galactorrhea, and other risk factors that may be responsible for galactorrhea. Preoperative examination included ultrasound of both breasts in all cases. In all cases, a recommended interval of at least 12 months between cessation of breastfeeding and the date of surgery was adhered to. Indication for augmentation mastopexy was Regnault's grade II ptosis and desire for volume augmentation in all three cases. Surgeries were performed by two board-certified surgeons (P.J. performed on cases 1 and 2 and M.P. performed on case 3). The procedure itself consisted of bilateral breast augmentation with silicone implants placed in a submuscular pocket with creation of dual plane type I and inframammary fold (IMF) reconstruction with Vicryl 0 running suture. The implant pocket below the pectoral major muscle was irrigated with Adams solution and betadine.

In the first case, Motiva Ergonomix 1 demi 310 mL (Establishment Labs Holdings, Alajuela, Costa Rica) were implanted bilaterally. In the second and third cases, moderate plus profile Mentor Xtra implants (Johnson and Johnson, NJ) were used. The volume in the second case was 325 mL bilaterally and in the third case 350 mL bilaterally.

This was followed by superior pedicle inverted T-scar mastopexy according to the principles of Mastopexy Made Applicable and Safer (MAMAS) surgical technique. 25 A fishtail-shaped wedge of lower pole tissues was removed perpendicularly until Scarpa's fascia was reached. This resection included glandular tissue located in the lower central part of the breast. Absorbable polyfilament sutures were used for subcutaneous tissue and glandular pillar approximation, absorbable monofilament stitches were used for subcutaneous and cutaneous sutures. Operating time did not extend over 90 minutes in any of the cases and drains were not used. Patients were dismissed on the first postoperative day. Checkup appointments were scheduled at 2 weeks, 6 weeks, 6 months, and 12 months postoperatively. All patients provided written consent for the publication of photographic materials and medical records for the purposes of this study.

#### Case Report 1

**Fig. 2:**
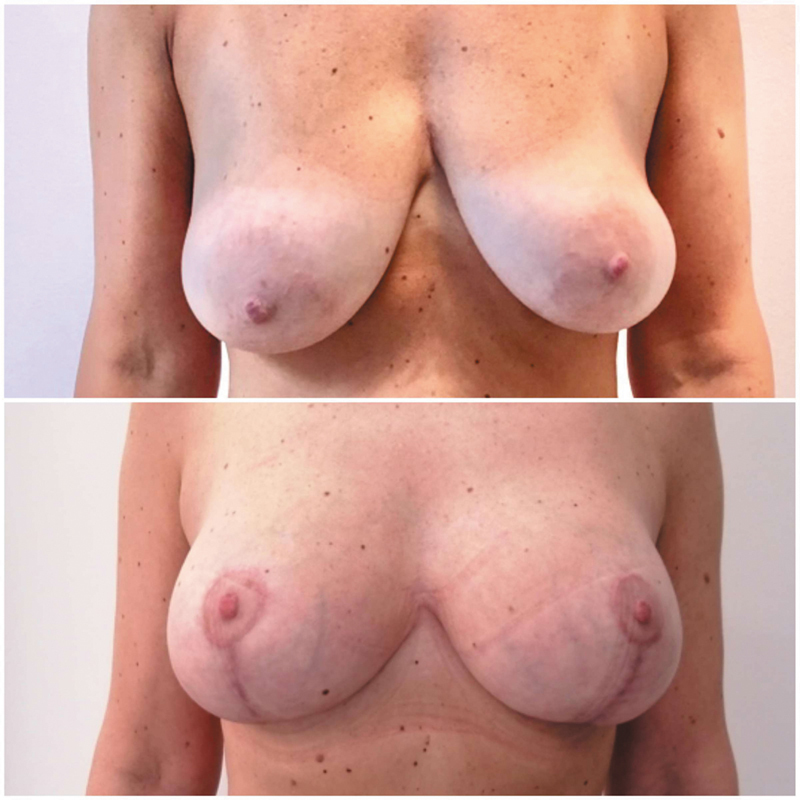
Case number 1: Preoperative photo and results after 2 months.

**Fig. 3:**
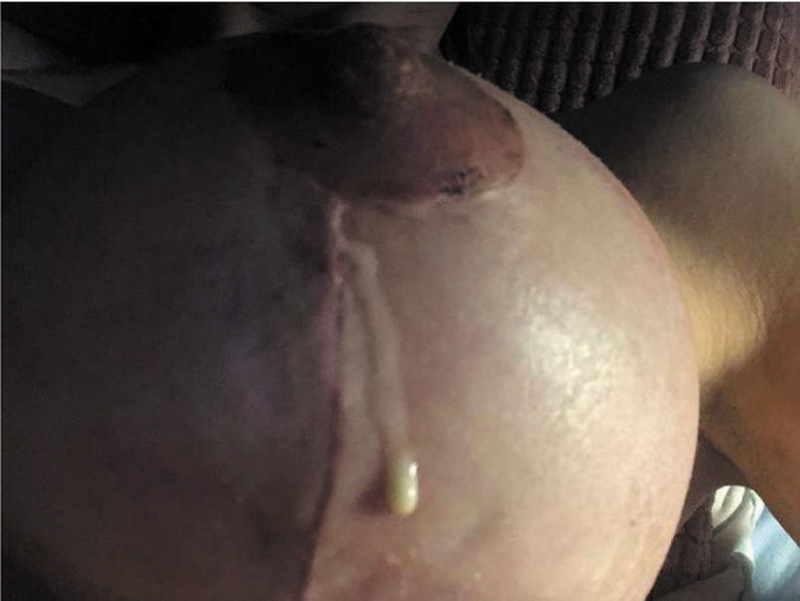
Case number 1: Excretion site of galactorrhea.

The first case was a 38-year-old woman who had two children and a history of hypothyroidism treated with levothyroxine. History of previous galactorrhea, surgery, or chest wall abnormalities was negative. Preoperative ultrasound did not show any breast abnormalities. The immediate postoperative course was insignificant and free from any other early-onset complications. Surgical outcome is shown in Fig. 2 . At the 2-week postoperative checkup, the patient did not present with any signs of galactorrhea. After the 16th postoperative day, she started to complain about slowly developing general pain in both breasts, uncommon for any of the usual postoperative muscle pain. Clinically, redness and swelling on both breasts were present, followed by secretion of white fluid from the vertical and circumareolar scar ( Fig. 3 ). Initial symptoms resembled an early surgical site infection; thus, the broad-spectrum antibiotics were administered. A microbiological swab ruled out a surgical site infection. A diagnosis of galactorrhea was made using Sudan IV stain test for fat droplets. Ultrasound of the breast showed only a small amount of liquid surrounding the breast glandular tissue. The patient was treated with cabergoline, and the secretion decreased immediately after initiation of the therapy and completely resolved in 2 weeks. At 12 months of follow-up, the breasts were soft and symmetric without any sign of capsular contracture or implant displacement.

#### Case Report 2

**Fig. 4:**
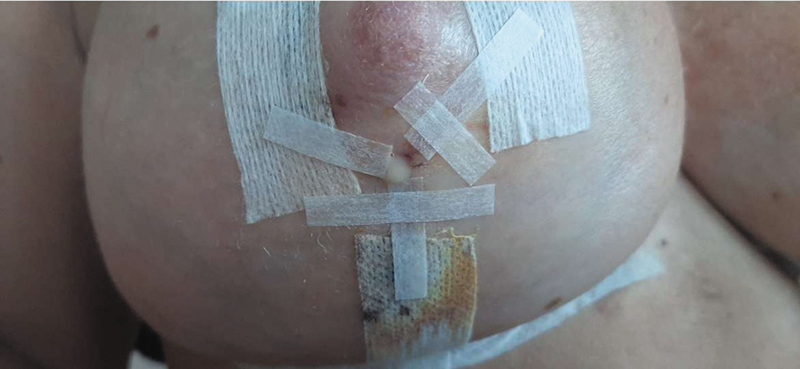
Case number 2: Excretion site of galactorrhea.

**Fig. 5:**
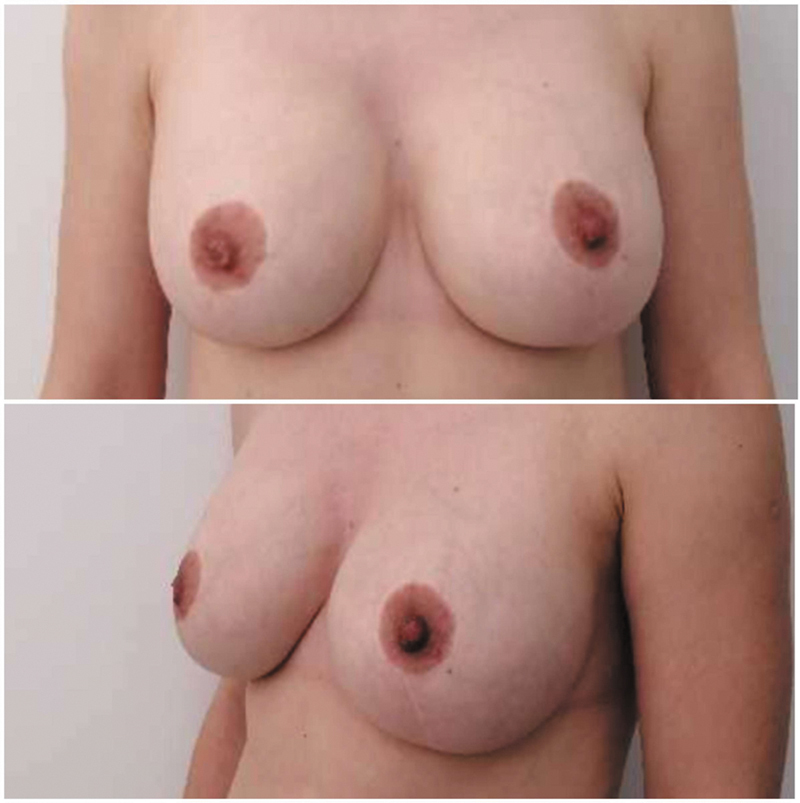
Case number 2: Long-term results after 12 months.

The second case was a 39-year-old female who had one child, but otherwise no relevant medical history. As in the first case, no perioperative or early postoperative complications occurred in the first 14 days following the surgery. On the 15th postoperative secretion around the nipple and from the T-junction ( Fig. 4 ) without surrounding tissue inflammation was noted and was again treated with broad-spectrum antibiotics. Culture of a microbiological swab was negative and galactorrhea was confirmed by the Sudan IV stain test. Galactocele formation was ruled out by ultrasound. The galactorrhea was treated with oral cabergoline with a good response, and no further surgical intervention was necessary. One-year follow-up showed good aesthetic outcome without any deformity, similar to the first case report ( Fig. 5 ).

#### Case Report 3

**Fig. 6:**
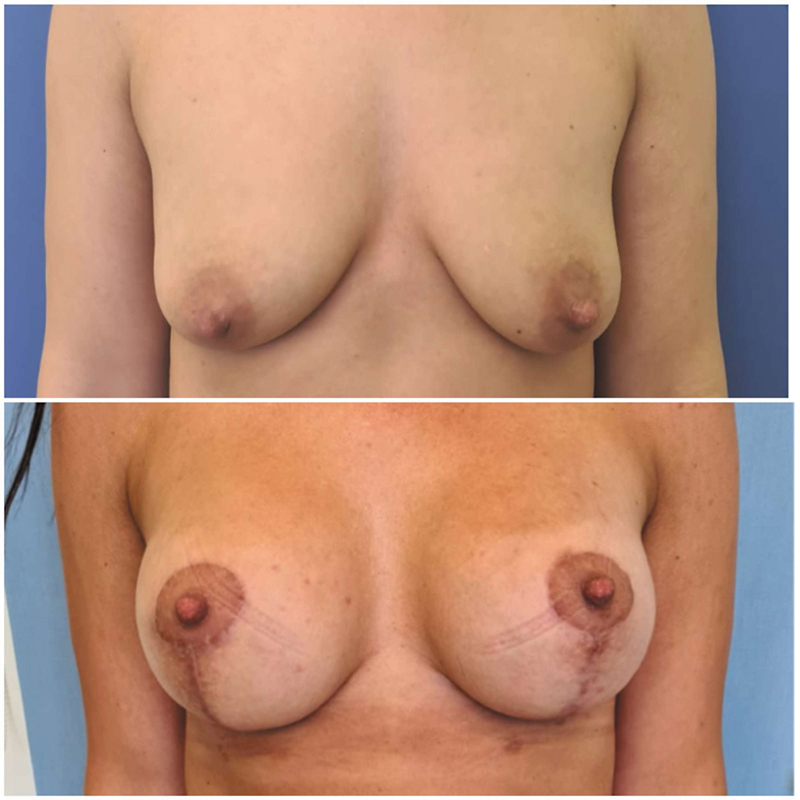
Case number 3: Pre- and postoperative results after 2 months (front view).

**Fig. 7:**
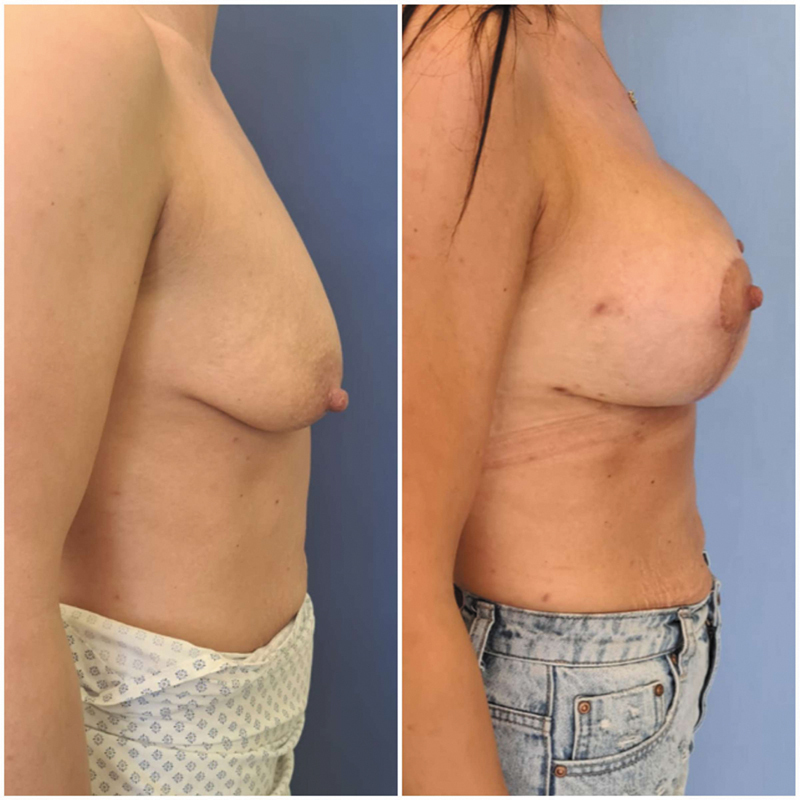
Case number 3: Pre- and postoperative results after 2 months (lateral view).

The third patient was a 32-year-old woman who had two children, a history of hypothyroidism treated with levothyroxine, and a mild form of myasthenia gravis not requiring treatment. The first signs of galactorrhea occurred 3 weeks after surgery. Again, as it resembled a surgical site infection and was initially treated with broad-spectrum antibiotics. Microbiological swab from the T-scar site was repeatedly negative. Ultrasound revealed a fluid collection in the deep glandular tissue surrounding the implant. Operative revision was necessary for bilateral dehiscence of the wound in the T-junction with implant exposure. Under general anesthesia, a small milk collection was evacuated, the secretion site was treated with electrocoagulation and drains were inserted. After the surgery, further milk secretion did not occur, and all clinical symptoms, including redness, swelling, and pain, resolved fully in the following days. In this case, due to thorough surgical revision and cauterization of the glandular tissue, milk secretion ceased, and the tension around the glands was released with drains. It did not recur in the early postoperative care, even after the patient's discharge. After removal of the drains, galactorrhea did not recur, and the patient healed without any further complications ( Figs. 6 and 7 ).

At 1-year follow-up, the breasts were soft, symmetric, without signs of capsular contracture, implant displacement, or galactorrhea recurrence. The patient, however, requested to undergo a further breast augmentation and is now waiting for this procedure.

**Fig. 8:**
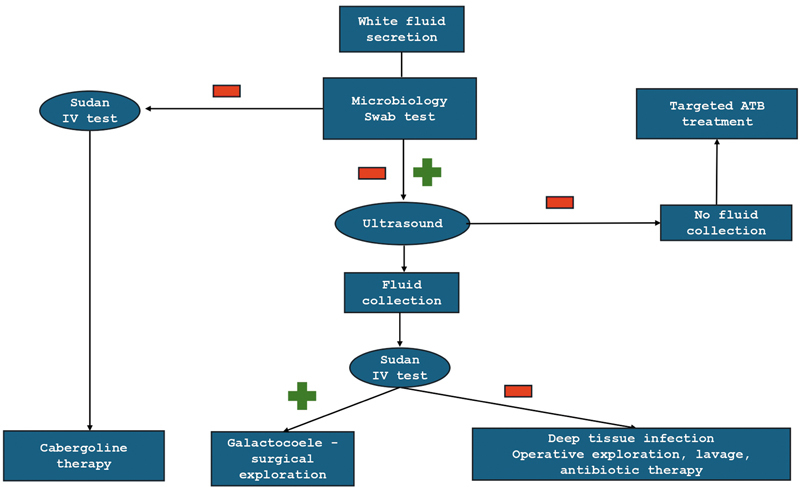
Treatment algorithm of galactorrhea.

The treatment algorithm of galactorrhea in our hands is described in Fig. 8 .

## Discussion

Galactorrhea is a rare but well-described complication following breast augmentation, breast reduction, or mastopexy. Incidence of postoperative galactorrhea has been reported to be between 0.8% and 0.96%. 21 23 Mastopexy augmentation combines both breast tissue reduction and breast augmentation; thus, manipulation with glandular parenchyma combined with direct chest wall irritation may increase the risk of galactorrhea. However, only very few cases were described in the literature following this type of procedure. 7 19

We report on the occurrence of galactorrhea in three cases of patients undergoing bilateral breast augmentation mastopexy. Revision surgery was required in one case due to wound dehiscence; the other two cases were managed with cabergoline alone.

Cabergoline works as a dopamine agonist and binds to D2 receptors of the pituitary gland and causing a decrease in plasmatic prolactin levels. It is used as a first-line medical therapy in patients with hyperprolactinemia, mainly in patients with prolactin-secreting adenomas of the pituitary gland, but might be used in other etiologies, such as postoperative galactorrhea. Cabergoline has a higher efficacy and tolerability compared with other dopamine agonists. Side effects are usually mild and include gastrointestinal symptoms, dizziness, and fatigue. 26

Hypothyroidism as a cause of hyperprolactinemia is a well-documented fact. Two of our patients were treated with levothyroxine for hypothyroidism. However, we do not suppose any relationship between hypothyroidism and galactorrhea in our patients, since they were both adequately treated according to their regular hormonal profile checkup with their endocrinologists.

### Incision and Secretion Sites

An interesting finding in our case reports was the location of the secretion site, in the middle third of the vertical scar around the NAC, and in the T-junction. This corresponds to the fact that the incision was made through the lactiferous duct when the NAC elevated using the superior pedicle, and the lower pole glandular tissue was removed.

Multiple secretion sites have been described in a case report by Rothkopf and Rosen in a patient undergoing the same surgical procedure as in our report. 19 Similar findings were also presented in cases of galactorrhea after breast reduction. Majdak-Paredes et al. 14 investigated galactorrhea in a postmenopausal woman after breast reduction with formation of a galactocele. Formation of neuromas of the fourth intercostal nerves, thus direct chest wall irritation was considered to be the main cause of galactorrhea. This theory was also supported by the fact that suppression of lactation occurred after amitriptyline administration, which is usually used for neuropathic pain. The authors also reviewed other studies of galactorrhea after mastopexy mostly linked to physiological postpartum lactation. Menéndez-Graíño et al. investigated galactorrhea after reduction mammaplasty in a patient 5 months after delivery. The patient experienced copious milk discharge from the periareolar and inframammary wounds. 15

In another case report, Vassilikos described galactorrhea in a patient 13 months following breast augmentation surgery, which spontaneously ceased after 2 weeks from the symptoms initiation without any treatment. 3 Basile and Basile presented a longitudinal retrospective case-series with patients after breast augmentation who suffered from bilateral nipple milk discharge. The incidence of galactorrhea after the periareolar approach was significantly higher. 8 However, this does not mean that inframammary approach is free from these complications. Guerra et al. presented a case report of galactorrhea and a galactocele in the IMF incision region, which had been until then considered to be a protective approach against galactorrhea. 11 These findings may also suggest that any profound manipulation of the NAC may produce lactation in some patients.

### Long-Term Results

In the context of long-term outcomes, in our case report, good long-term outcomes were achieved in all cases. The majority of studies have documented either satisfactory or excellent results in appropriately managed patients. However, a notable observation emerges from five studies, 2 7 9 13 24 where long-term complications manifested as objectively suboptimal aesthetic outcomes, diverging from patient satisfaction rates. Noteworthy instances include two case reports detailing breast reduction procedures marked by prolonged wound healing, 13 leading to skin and nipple necrosis, 2 necessitating subsequent surgical interventions and resulting in substantial scar formation.

Of particular interest is the occurrence of capsular contracture, which was documented in only one case, occurring 2 years after breast augmentation with a double lumen prosthesis. In the comprehensive study conducted by Caputy and Flowers, 9 instances of breast asymmetry and implant deflation were documented. The correlation between implant deflation and prior galactorrhea raises intriguing questions warranting further investigation.

Ayestaray et al. reported postoperative double bubble deformity in the right breast after augmentation mammaplasty. 7 Formation of a large milk deposit in the implant pocket may cause its enlargement or disintegration IMF. Late complications with a possible link to galactorrhea are summarized in Table 1 .

**Table 1:** Number of studies where late complications related to galactorrhea occurred

Number	Study	Surgery	Implant	Complications
1	Bentley et al. 2 (2003)	Breast reduction	–	Right breast skin and nipple necrosis, secondary surgeries required
2	Arnon et al. 13 (2006)	Breast reduction	–	Prolongs wound healing, secondary surgeries required
3	Caputy and Flowers (1994) 9	Breast augmentation	Double-lumen prosthesis	Capsular contraction bilateral, deflation right side
4	Caputy and Flowers (1994) 9	Breast augmentation	Double-lumen prosthesis	Deflation
5	Caputy and Flowers (1994) 9	Breast augmentation	Double-lumen prosthesis	Asymmetry
6	Ayestaray et al. (2011) 7	Breast augmentation + mastopexy	Round implant	Double bubble deformity
7	Tung and Carr (2011) 24	Breast augmentation	Round implant	Asymmetry

### Conclusion

Despite being a rare complication, galactorrhea should not be underestimated.

Without proper treatment, it may lead to an increased risk of more severe complications. Our patients' long-term results did not show any late complications regardless of the secretion site. In all cases, good aesthetic results with high patient satisfaction were achieved.

## References

[1] Huang W Molitch M E Evaluation and management of galactorrhea Am Fam Physician 2012 85 11 1073 1080 22962879

[2] Bentley M Ghali S Asplund O A Galactorrhoea causing severe skin breakdown and nipple necrosis following breast reduction Br J Plast Surg 2004 57 07 682 684 15380704 10.1016/j.bjps.2004.04.023

[3] Vassilikos C Lactation: a rare, late complication of augmentation mammaplasty Aesthet Surg J 2004 24 05 449 450 19336194 10.1016/j.asj.2004.07.007

[4] Torre D L Falorni A Pharmacological causes of hyperprolactinemia Ther Clin Risk Manag 2007 3 05 929 951 18473017 PMC2376090

[5] Atluri S Sarathi V Goel A Boppana R Shivaprasad C Etiological profile of galactorrhoea Indian J Endocrinol Metab 2018 22 04 489 493 30148095 10.4103/ijem.IJEM_89_18 PMC6085969

[6] Fachi M M de Deus Bueno L de Oliveira D C da Silva L L Bonetti A F Efficacy and safety in the treatment of hyperprolactinemia: A systematic review and network meta-analysis J Clin Pharm Ther 2021 46 06 1549 1556 34137053 10.1111/jcpt.13460

[7] Ayestaray B Dudrap E Chaibi A Galactorrhea after aesthetic breast augmentation with silicone implants: report of two cases and management of postoperative galactorrhea Aesthetic Plast Surg 2011 35 03 408 413 20927519 10.1007/s00266-010-9595-6

[8] Basile F V Basile A R Diagnosis and management of galactorrhea after breast augmentation Plast Reconstr Surg 2015 135 05 1349 1356 25919249 10.1097/PRS.0000000000001156

[9] Caputy G G Flowers R S Copious lactation following augmentation mammaplasty: an uncommon but not rare condition Aesthetic Plast Surg 1994 18 04 393 397 7817889 10.1007/BF00451346

[10] Chun Y S Taghinia A Hyperprolactinemia and galactocele formation after augmentation mammoplasty Ann Plast Surg 2009 62 02 122 123 19158518 10.1097/SAP.0b013e31817d8832

[11] Guerra M Codolini L Cavalieri E Redi U Ribuffo D Galactocele after aesthetic breast augmentation with silicone implants: an uncommon presentation Aesthetic Plast Surg 2019 43 02 366 369 30456639 10.1007/s00266-018-1266-z

[12] Hartley J H Jr Schatten W E Postoperative complication of lactation after augmentation mammaplasty Plast Reconstr Surg 1971 47 02 150 153 5107582 10.1097/00006534-197102000-00009

[13] Arnon O Mendes D Winkler E Tamir J Orenstein A Haik J Galactorrhea complicating wound healing following reduction mammaplasty Aesthet Surg J 2006 26 03 300 301 19338911 10.1016/j.asj.2006.03.002

[14] Majdak-Paredes E J Shafighi M During V Sterne G D An unusual case of galactorrhea in a postmenopausal woman complicating breast reduction J Plast Reconstr Aesthet Surg 2009 62 04 542 546 18023264 10.1016/j.bjps.2007.10.019

[15] Menéndez-Graíño F Pena Fernández C Burrieza P I Galactorrhea after reduction mammaplasty Plast Reconstr Surg 1990 85 04 645 646 10.1097/00006534-199004000-00047 2315414

[16] Qureshi A A Myckatyn T M Tenenbaum M M Mastopexy and mastopexy-augmentation Aesthet Surg J 2018 38 04 374 384 29365038 10.1093/asj/sjx181

[17] Duarte Junior G Duarte F C Cervantes A Mastopexy with an implant and the making of a horizontal flap of the upper pedicle, simulating an internal bra Aesthetic Plast Surg 2022 46 01 11 21 10.1007/s00266-021-02481-3 34309692

[18] Gupta V Yeslev M Winocour J Aesthetic breast surgery and concomitant procedures: incidence and risk factors for major complications in 73,608 cases Aesthet Surg J 2017 37 05 515 527 28333172 10.1093/asj/sjw238

[19] Rothkopf D M Rosen H M Lactation as a complication of aesthetic breast surgery successfully treated with bromocriptine Br J Plast Surg 1990 43 03 373 375 2350650 10.1016/0007-1226(90)90095-h

[20] Yang E J Lee K T Pyon J K Bang S I Treatment algorithm of galactorrhea after augmentation mammoplasty Ann Plast Surg 2012 69 03 247 249 22214792 10.1097/SAP.0b013e31822af880

[21] Onishi T Miyai K Aono T Shioji T Yamamoto T Primary hypothyroidism and galactorrhea Am J Med 1977 63 03 373 378 409288 10.1016/0002-9343(77)90275-3

[22] Meier C Christ-Crain M Guglielmetti M Huber P Staub J J Müller B Prolactin dysregulation in women with subclinical hypothyroidism: effect of levothyroxine replacement therapy Thyroid 2003 13 10 979 985 14611708 10.1089/105072503322511391

[23] Karimi H Nourizad S Momeni M Rahbar H Momeni M Farhadi K Burns, hypertrophic scar and galactorrhea J Inj Violence Res 2013 5 02 117 119 23456048 10.5249/jivr.v5i2.314 PMC3683415

[24] Tung A Carr N Postaugmentation galactocele: a case report and review of literature Ann Plast Surg 2011 67 06 668 670 21346529 10.1097/SAP.0b013e3182069b3c

[25] Payer J Chalkidis N Polackova P Patzelt M MAMAS (mastopexy-augmentation made applicable and safer): A standardized template of pre-operative marking and step-by-step surgical procedure JPRAS Open 2024 40 293 304 38708383 10.1016/j.jpra.2024.03.007 PMC11070225

[26] Petersenn S Fleseriu M Casanueva F F Diagnosis and management of prolactin-secreting pituitary adenomas: A Pituitary Society international Consensus Statement Nat Rev Endocrinol 2023 19 12 722 740 37670148 10.1038/s41574-023-00886-5

